# Efficacy and safety of preoperative intranasal dexmedetomidine in adults: a systematic review and meta-analysis of randomized controlled trials

**DOI:** 10.1016/j.bjane.2026.844820

**Published:** 2026-09-03

**Authors:** Vitaliy Voznyy, Mouad Elganga, Leya Tawfik, Tony Tan, Sammie Yu, Ellene Yan, Aparna Saripella, Marina F. Englesakis, Frances Chung

**Affiliations:** a University of Toronto, Temerty Faculty of Medicine, Toronto, ON, Canada; b University of Toronto, Temerty Faculty of Medicine, Institute of Medical Science, Toronto, ON, Canada; c University of Toronto, University Health Network, Toronto Western Hospital, Department of Anesthesia and Pain Management, Toronto, ON, Canada; d University Health Network, Library & Information Services, Toronto, ON, Canada

**Keywords:** Administration, Intranasal, Adult, Anesthesia, General, Dexmedetomidine, Hemodynamics, Meta-analysis as topic

## Abstract

Background: Airway manipulation during General Anesthesia (GA) can produce sympathetic activation and hemodynamic instability. Intravenous (IV) dexmedetomidine attenuates these responses but may cause bradycardia or hypotension. Intranasal (IN) administration is a noninvasive alternative with uncertain evidence in adults. We evaluated preoperative IN dexmedetomidine in adult patients undergoing GA.

Methods: We performed a PROSPERO-registered (CRD420251250492) systematic review and meta-analysis of randomized trials. MEDLINE, Embase, CENTRAL, and CDSR were searched from inception to November 26, 2025. IN dexmedetomidine versus control and IN vs. IV dexmedetomidine were assessed separately. Random-effects models pooled continuous outcomes as MDs/SMDs and adverse events as RRs. Risk of bias was assessed using RoB2 and certainty of evidence using GRADE.

Results: Across 25 trials including 1,968 patients, IN dexmedetomidine was associated with deeper sedation (SMD = 0.76; 95% CI: 0.18–1.34) and reduced heart rate and mean arterial pressure at induction, intubation, and during the early intraoperative period compared with control (MD range: -12.7 to -13.4 bpm and -7.8 to -10.7 mmHg, respectively). Rates of bradycardia and hypotension were similar between IN dexmedetomidine and control groups. Compared with IV dexmedetomidine, IN administration resulted in lighter sedation (SMD = -0.54; 95% CI: -1.08 to -0.01), with similar hemodynamic effects, while bradycardia and hypotension occurred less frequently.

Conclusion: Preoperative IN dexmedetomidine may improve sedation and blunt peri-induction hemodynamic responses compared with controls, with broadly similar hemodynamic effects and fewer observed cardiovascular adverse events versus IV administration. However, certainty was low or very low for several efficacy outcomes, and larger well-designed trials are needed.

## Introduction

Perioperative sympathetic activation during induction of General Anesthesia (GA) and tracheal intubation remains a major contributor to hemodynamic instability, myocardial stress, and perioperative complications.^1^ Excessive tachycardia and hypertension during airway manipulation are associated with increased myocardial oxygen demand, while excessive anesthetic depth or aggressive pharmacologic attenuation can precipitate hypotension and bradycardia.^2^ Given the vulnerability of surgical patients to perioperative neurocognitive disorders, achieving adequate anxiolysis and blunting of stress responses without compromising hemodynamic or respiratory safety remains a central challenge in anesthetic practice.^3^

Dexmedetomidine, a selective α2-adrenergic receptor agonist, has emerged as a useful adjunct due to its sedative, anxiolytic, and sympatholytic properties.^4^ Unlike many commonly used sedatives, it produces effective sedation with limited respiratory depression, making it particularly appealing during the peri-induction period.^4^ Intravenous (IV) dexmedetomidine has been studied extensively, demonstrating efficacy in attenuating hemodynamic responses to laryngoscopy and intubation, reducing perioperative Heart Rate (HR) and blood pressure variability, and improving perioperative sedation quality.^5^, ^6^, ^7^ IV administration is associated with clinically significant bradycardia and hypotension, and may be undesirable or impractical in select perioperative settings or in patients with needle-related anxiety.^8^^,^^9^

Intranasal (IN) administration of dexmedetomidine represents a noninvasive alternative, offers relatively high bioavailability, and provides pain-free gradual systemic absorption.^10^ Absorption across the nasal mucosa bypasses first-pass hepatic metabolism, yielding a bioavailability of approximately 65% in adults with a more gradual rise in plasma concentration than the IV route.^11^ This pharmacokinetic profile can minimize reflex bradycardia and hemodynamic instability associated with IV bolus administration. Peak sedative effect following IN dexmedetomidine occurs approximately 45 to 60 minutes after administration,^11^ aligning with typical preoperative holding periods and supporting its use as preanesthetic medication. While IN dexmedetomidine is well established in pediatric anesthesia and procedural sedation,^12^^,^^13^ robust evidence in adults remains limited, and clinical adoption in adult practice has lagged accordingly.^14^, ^15^, ^16^ To date, the safety profile of IN versus IV dexmedetomidine in adult surgical patients has not been directly compared in a meta-analysis, despite the differing pharmacokinetic behavior of the two routes.

The objective of this systematic review and meta-analysis of Randomized Controlled Trials (RCTs) was to evaluate preoperative IN dexmedetomidine in adults having surgery under GA, compared with IV dexmedetomidine and control, across various clinically relevant domains: depth of sedation, attenuation of the hemodynamic response to intubation, postoperative analgesic requirements, and adverse events.

## Methods

This systematic review and meta-analysis was registered with the International Prospective Register of Systematic Reviews (PROSPERO; CRD420251250492) and reported in accordance with the Preferred Reporting Items for Systematic Reviews and Meta-Analyses (PRISMA) guidelines.^17^ The two pairwise comparisons, IN dexmedetomidine versus control and IN versus IV dexmedetomidine, were prespecified in the PROSPERO protocol. The protocol also prespecified the relevant outcome domains, including perioperative hemodynamics, sedation, opioid consumption, postoperative nausea and vomiting, and adverse events such as bradycardia and hypotension. The protocol did not explicitly classify outcomes as primary or secondary. For the present report, depth of sedation, perioperative hemodynamics, bradycardia, and hypotension were prioritized as primary outcomes because they directly reflect the intended premedication effects and principal cardiovascular safety concerns of dexmedetomidine. Propofol consumption, postoperative opioid consumption, Post-Anesthesia Care Unit (PACU) time, and postoperative nausea and vomiting (PONV) were considered secondary outcomes. The protocol included additional outcome domains, but they were not pooled due to insufficient data.

### Literature search strategy

A comprehensive literature search was conducted by an information specialist (MFE) using the Ovid platform, encompassing MEDLINE, MEDLINE ePubs and In-Process Citations (daily), Embase Classic and Embase, the Cochrane Central Register of Controlled Trials, and the Cochrane Database of Systematic Reviews. Searches were completed from inception to November 26, 2025. The search strategy was developed in accordance with the Cochrane Handbook,^18^ and the Methodological Expectations for Cochrane Intervention Reviews (MECIR).^19^ Preliminary scoping searches and full-text reviews were undertaken to identify relevant keywords and controlled vocabulary, including MeSH terms for MEDLINE and EMTREE terms for Embase. The Yale MeSH Analyzer was used to assist in the identification of appropriate subject headings and text-word terms.^20^ The final search strategy combined controlled vocabulary and text-word terms related to preoperative IN dexmedetomidine, inpatient surgery, general anesthesia, perioperative or postoperative outcomes, and RCT study designs. Searches were limited to studies published in English involving adult human populations, and conference abstracts and other non-peer-reviewed materials were excluded. The MEDLINE search strategy is provided in Table S1. For eligible studies where full texts were unavailable, corresponding authors were contacted to request access.

### Study selection criteria

The inclusion criteria were: 1) Adults (≥ 18 years) having inpatient surgery under GA; 2) Preoperative IN dexmedetomidine administered; and 3) RCTs that included a comparator arm, defined as either a control group (placebo or standard care) or an IV dexmedetomidine group. The exclusion criteria included non-randomized study designs, case reports, case series, and cross-sectional studies.

Two independent reviewers (LT, TT) performed title and abstract screening on Covidence. Further, two reviewers (ME, LT) conducted full-text screening independently. Conflicts were resolved by a third reviewer (VV). No automation tools were used in the study selection process. On July 26, 2026, the publication status of all included studies and key references was checked in PubMed, where indexed, the Retraction Watch Database, Crossmark/Crossref, and the relevant journal or publisher websites. No retractions, withdrawals, expressions of concern, or published corrections were identified.

### Data extraction

Four independent reviewers (VV, LT, TT, SY) extracted data in duplicate into a standardized form. Discrepancies were resolved by a fifth reviewer (ME). The extraction form included study-level information (year of publication, title, country, type of surgery, sample size for each group IN dexmedetomidine vs. IV dexmedetomidine vs. control, dexmedetomidine dose, and time of administration) and demographics (age, Body Mass Index BMI, and male percentage). Outcomes were recorded regarding depth of sedation, perioperative Hemodynamics (HR and MAP), adverse events (bradycardia, hypotension, and PONV), postoperative opioid consumption, PACU time, and propofol consumption.

### Risk of bias assessment

The risk of bias of studies included in the pooled analysis was independently evaluated by two reviewers (ME, VV) using the Revised Cochrane Risk of Bias tool for randomized trials (RoB2).^21^ Risk of bias was evaluated across five domains: randomization process, deviations from intended interventions, missing outcome data, outcome measurement, and selection of reported results. For each study, individual domains were classified as low, some concerns, or high risk of bias. The overall risk of bias for each study was determined by the highest concern level across the domains. Disagreements in risk-of-bias judgments were resolved by a third reviewer (LT).

### Statistical analysis

Continuous outcomes (sedation scores, HR, and MAP) were pooled as Mean Differences (MD) or Standardized Mean Differences (SMD) using Random‐Effects Models (REML). Sedation scales with inverse directionality were harmonized prior to analysis so that positive SMD represented deeper sedation with IN dexmedetomidine. Binary adverse outcomes (bradycardia, hypotension) were analyzed as Risk Ratios (RR). A minimum of two studies was required for meta-analysis. Estimates were presented with 95% confidence intervals, pooled heterogeneity (*I*^2^, τ²), Cochran’s *Q*, and random-effects p-values. All analyses were conducted using the meta^22^ and metafor^23^ packages in R (4.5.1); p-value of < 0.05 was considered statistically significant. Sensitivity analyses using leave-one-out procedures were performed for all outcomes with at least three studies. Subgroup analyses by type of surgery, dexmedetomidine dose or timing, and outcome measurement scale were prespecified in the protocol. However, the number of studies contributing to each pooled outcome was insufficient to support meaningful subgroup analysis, and these were therefore not performed.

### GRADE appraisal of evidence

The certainty of evidence for each outcome was assessed according to the Grading of Recommendations, Assessment, Development, and Evaluation (GRADE) guidelines.^24^ Certainty was evaluated across five domains: risk of bias, imprecision, inconsistency, indirectness, and publication bias. The summary table was created using the GRADEpro Guideline Development Tool.^25^

## Results

### Study selection

**Figure 1:**
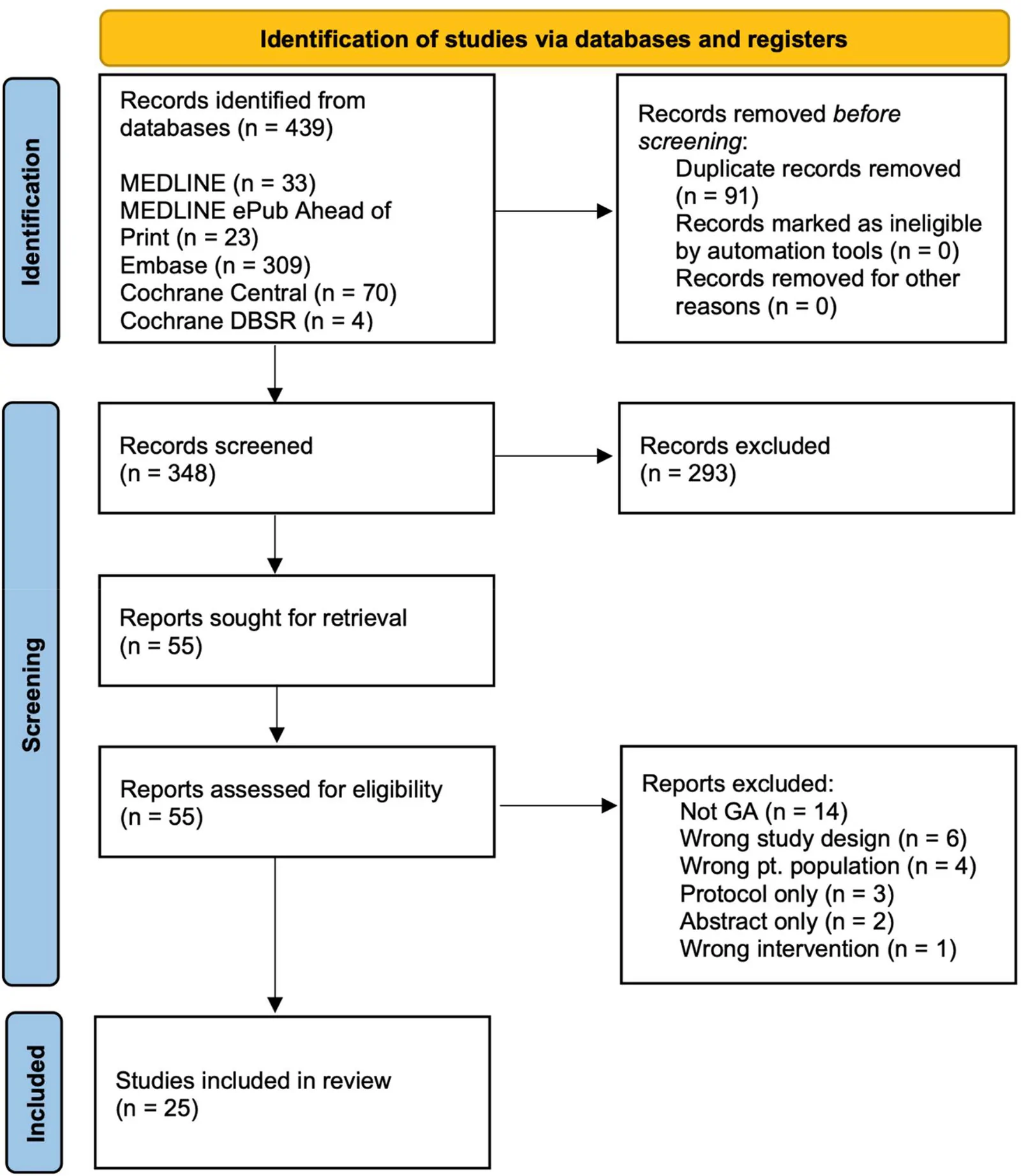
PRISMA flow diagram.

The literature search identified 439 records. After removal of 91 duplicates, 348 records were screened by title and abstract. Of these, 293 records were excluded. Full-text review was conducted for 55 studies, of which 30 were excluded. A total of 25 RCTs^14^, ^15^, ^16^^,^^26^, ^27^, ^28^, ^29^, ^30^, ^31^, ^32^, ^33^, ^34^, ^35^, ^36^, ^37^, ^38^, ^39^, ^40^, ^41^, ^42^, ^43^, ^44^, ^45^, ^46^, ^47^ met eligibility criteria and were included in the systematic review and meta-analysis. Figure 1 presents the PRISMA flow diagram of study selection.

### Study characteristics

The 25 included RCTs enrolled 1,968 adult surgical patients undergoing a range of inpatient procedures. Fourteen studies compared IN dexmedetomidine with control,^14^, ^15^, ^16^^,^^26^^,^^27^^,^^29^^,^^31^^,^^33^^,^^37^^,^^38^^,^^41^^,^^45^, ^46^, ^47^ nine compared IN with IV dexmedetomidine,^28^^,^^32^^,^^34^, ^35^, ^36^^,^^39^^,^^40^^,^^43^^,^^44^ and two studies included both IN and IV dexmedetomidine arms alongside a control group.^30^^,^^42^ Among trials with a control arm, comparators were placebo controls, usually intranasal or nebulized 0.9% saline, except for one trial that used oral alprazolam as an active control.^41^ Individual study sample sizes ranged from 29 to 159 participants (Table 1). Mean age and BMI across studies were 49 ± 16 years and 25 ± 5 kg.m^-2^, respectively. The proportion of male participants varied across studies, ranging from 0% in a gynecologic surgery population to 78% in an Ear, Nose, and Throat (ENT) surgery population. The included studies were predominantly conducted in India (n = 12), followed by China (n = 10), with fewer studies originating from Egypt (n = 2) and Iran (n = 1).

**Table 1:** Characteristics of included studies.

Study	Sample Size	Mean Age (y)	Mean BMI (kg.m^-2^)	Male %	Surgery Type	Intranasal Dexmedetomidine Dose	Primary Comparator	Administration Device
Gupta 2025, India	75	31.7 ± 9.1	NR	56.0	Laparoscopy	1 μg.kg^-1^	Control and IV	Nasal drops
Singh 2025, India	120	42.4 ± 12.1	24.9 ± 2.9	58.3	Neurosurgery	1 μg.kg^-1^	IV	Nasal drops
Wang 2025, China	159	48.7 ± 18.5	23.6 ± 2.6	61.6	ENT	75–100 μg	Control	Nasal spray (metered)
Choudhary 2024, India	120	NR	NR	31.7	Mixed	0.75 μg.kg^-1^	IV	Nasal drops
Fang 2024, China	100	67.5 ± 3.9	23.7 ± 3.0	59.0	Cardiac	0.3 μg.kg^-1^	Control	Nasal drops
He 2024, China	80	68.8 ± 2.9	22.1 ± 1.9	0.0	Gynecology	1.5 μg.kg^-1^	Control	Nasal drops
Kohaf 2024, Egypt	60	44.3 ± 11.0	25.6 ± 2.7	78.3	ENT	1 μg.kg^-1^	IV	Nasal drops
Mohammed 2024, Egypt	70	40.6 ± 10.7	26.0 ± 3.9	54.3	ENT	1 μg.kg^-1^	IV	Nasal drops
Rani 2024, India	90	41.5 ± 11.7	25.3 ± 2.9	54.4	NR	1 μg.kg^-1^	Control	Nebulizer
Yang 2024, China	60	50.7 ± 5.2	23.1 ± 2.6	38.3	NR	2.5 μg.kg^-1^	Control	Nasal drops
Kaila 2023, India	80	35.4 ± 7.9	NR	53.8	Mixed	1 μg.kg^-1^	Control	Nebulizer
M 2023, India	106	39.8 ± 12.3	NR	NR	Mixed	1 μg.kg^-1^	IV	Nasal drops
Niu 2023, China	99	70.4 ± 6.5	24.2 ± 4.2	47.5	Spine	1 μg.kg^-1^	IV	Nasal drops
Safavi 2022, Iran	59	41.89 ± 13.97	NR	32.9	NR	1 μg.kg^-1^	Control	NR
Singh 2022, India	120	41.7 ± 10.4	25.0 ± 3.5	51.7	Mixed	1 μg.kg^-1^	IV	Nebulizer
Suryawanshi 2022, India	60	43.4 ± 13.3	NR	51.7	Mixed	1 μg.kg^-1^	Control	Nebulizer
Wu 2022, China	29	41.6 ± 12.2	22.1 ± 1.9	44.8	ENT	1 μg.kg^-1^	IV	MAD
Zeng 2022, China	72	39.3 ± 11.3	22.2 ± 2.1	40.3	NR	2.5 μg.kg^-1^	Control	Nasal drops
Kochhar 2021, India	60	34.5 ± 11.6	NR	55.0	Mixed	1 μg.kg^-1^	Control	Nasal drops
Niyogi 2019, India	70	41.4 ± 11.7	22.9 ± 2.1	58.6	Spine	100 μg	IV	Nasal drops
Niyogi 2018, India	70	41.4 ± 11.7	22.8 ± 1.7	58.6	Spine	1 μg.kg^-1^	Control	Nasal drops
Lu 2016, China	81	44.7 ± 9.7	22.7 ± 4.2	35.8	Suspension Laryngoscopy	1 μg.kg^-1^	Control	Nasal drops
Qiao 2016, China	60	49.0 ± 12.3	23.4 ± 3.3	60.0	ENT	2 μg.kg^-1^	Control	MAD
Wu 2016, China	80	47.3 ± 4.7	NR	0.0	Gynecology	1 μg.kg^-1^	Control and IV	NR
Jayaraman 2013, India	40	NR	46.9 ± 7.0	NR	Bariatric	1 μg.kg^-1^	Control	Nasal drops

ENT, Ear Nose and Throat; IV, Intravenous; MAD, Mucosal Atomization Device; NR, Not Reported.

Surgical procedures included Ear, Nose, and Throat (ENT), neurological, spine, orthopedic, cardiac, gynecologic, bariatric, suspension laryngoscopy, and mixed elective surgery. IN dexmedetomidine dosing strategies varied. Most trials administered 1 μg.kg^-1^, while others used lower weight-based doses (0.3‒0.75 μg.kg^-1^), higher doses up to 2‒2.5 μg.kg^-1^, or fixed dosing regimens ranging from 50 to 100 μg. Table 2, Table 3 provide a summary of all pooled effects.

**Table 2:** Pooled effects for outcomes in intranasal dexmedetomidine compared with control.

Outcome	Effect on IN Group (95% CI)	n studies (patients)	p-value	*I*^2^	GRADE Certainty of Evidence
Sedation Score	SMD **0.76 Higher** (0.18‒1.34)	5 (393)	0.010	87.6%	Low
HR					
Baseline	MD **0.38 Lower** (−2.57‒1.80)	8 (568)	0.730	50.8%	Low
Induction	MD **13.37 Lower** (−18.36 to -8.39)	2 (110)	< 0.001	9.2%	High
Intubation	MD **12.73 Lower** (−17.66 to -7.80)	7 (508)	< 0.001	86%	Very Low
10 Minutes	MD **13.15 Lower** (−18.02 to -8.27)	4 (249)	< 0.001	77.4%	Low
MAP					
Baseline	MD **1.05 Higher (**-0.97‒3.08)	7 (508)	0.309	42.9%	Moderate
Induction	MD **10.65 Lower** (−16.56 to -4.75)	2 (110)	< 0.001	60.3%	Moderate
Intubation	MD **10.44 Lower** (−15.37 to -5.52)	7 (508)	< 0.001	85.3%	Very Low
10 Minutes	MD **7.77 Lower** (−14.56 to -0.98)	4 (249)	0.025	87.5%	Very Low
Bradycardia Incidence	RR **1.15** (0.64‒2.04)	5 (433)	0.645	0%	High
Hypotension Incidence	RR **1.09** (0.61‒1.96)	6 (493)	0.769	7.9%	High

IN, Intranasal Dexmedetomidine; HR, Heart Rate; MAP, Mean Arterial Pressure; SMD, Standardized Mean Difference; MD, Mean Difference; RR, Risk Ratio; CI, Confidence Interval; *I*^2^, Heterogeneity statistic; GRADE, Grading of Recommendations, Assessment, Development and Evaluation.

**Table 3:** Pooled effects for outcomes in intranasal dexmedetomidine compared with intravenous dexmedetomidine.

Outcome	Effect on IN Group (95% CI)	n studies	p-value	*I*^2^	GRADE Certainty of Evidence
Sedation Score	SMD **0.54 Lower** (−1.08 to -0.01)	4 (303)	0.047	78.7%	Low
HR					
Baseline	MD **0.14 Lower** (−3.41‒3.14)	6 (495)	0.934	65.9%	Low
Induction	MD **1.41 Higher** (−0.36‒3.17)	5 (466)	0.118	0%	High
Intubation	MD **2.44 Higher** (−1.08‒5.96)	4 (360)	0.174	62%	Moderate
10 Minutes	MD **4.09 Higher** (−0.18‒8.37)	6 (449)	0.061	87.4%	Low
MAP					
Baseline	MD **0.10 Higher** (−2.17‒2.37)	7 (555)	0.933	49.6%	Moderate
Induction	MD **0.81 Lower** (−2.44‒0.82)	4 (360)	0.332	0%	High
Intubation	MD **1.83 Higher** (−0.02‒3.68)	5 (466)	0.052	40.4%	Moderate
10 Minutes	MD **0.69 Higher** (−1.80‒3.18)	7 (555)	0.588	71.6%	Moderate
Bradycardia Incidence	RR **0.31** (0.11‒0.87)	4 (363)	0.026	0%	High
Hypotension Incidence	RR **0.15** (0.03‒0.79)	2 (293)	0.026	0%	High

IN, Intranasal Dexmedetomidine; HR, Heart Rate; MAP, Mean Arterial Pressure; SMD, Standardized Mean Difference; MD, Mean Difference; RR, Risk Ratio; CI, Confidence Interval; *I*^2^, Heterogeneity statistic; GRADE, Grading of Recommendations, Assessment, Development and Evaluation.

### Clinical effects of IN dexmedetomidine compared with control

**Figure 2:**
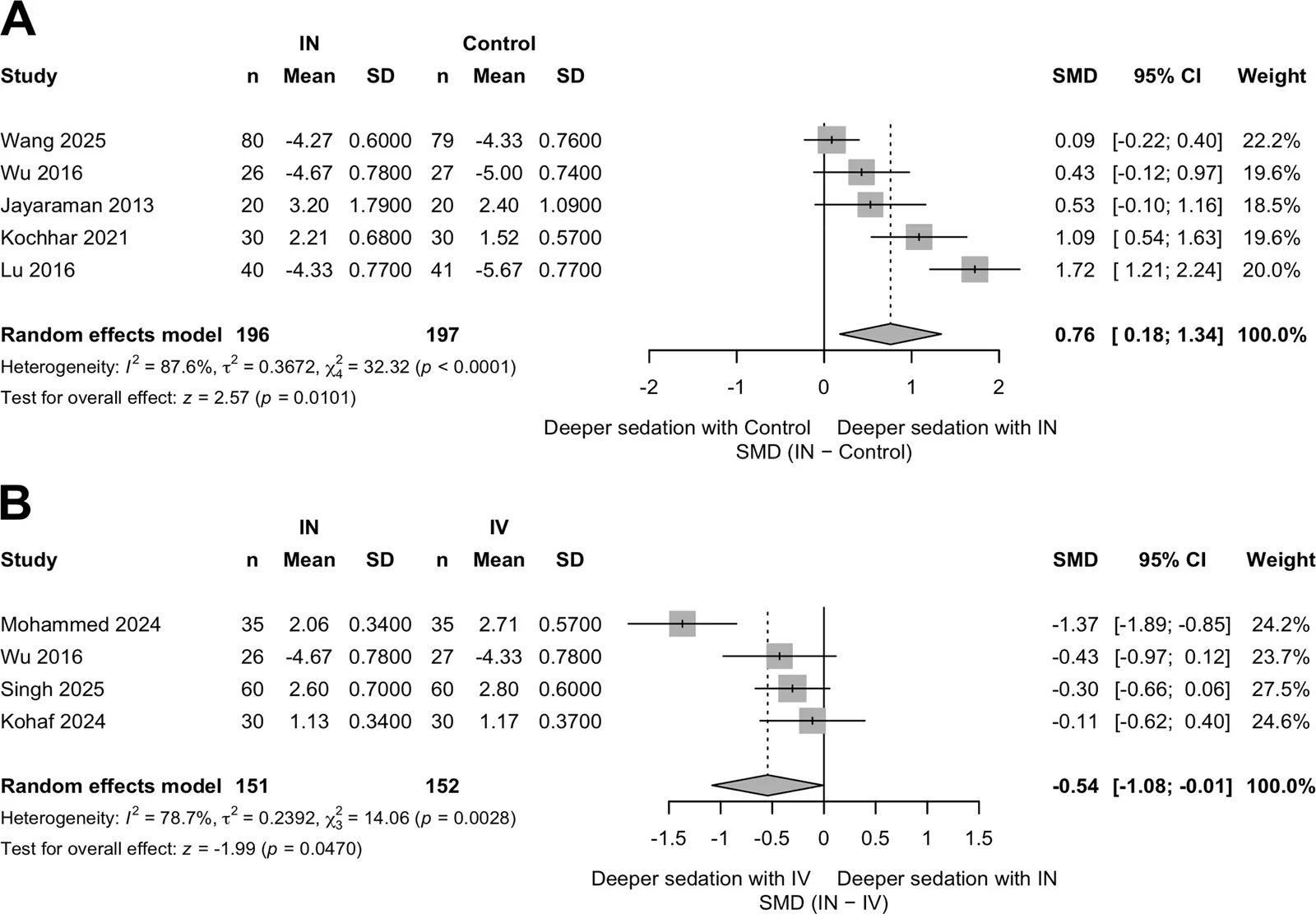
Forest plots for sedation score post-medication comparing Intranasal (IN) dexmedetomidine with (A) control and (B) Intravenous (IV) dexmedetomidine. The effect measure calculated was standardized mean difference. Positive values reflect deeper sedation with IN relative to the comparator.

**Figure 3:**
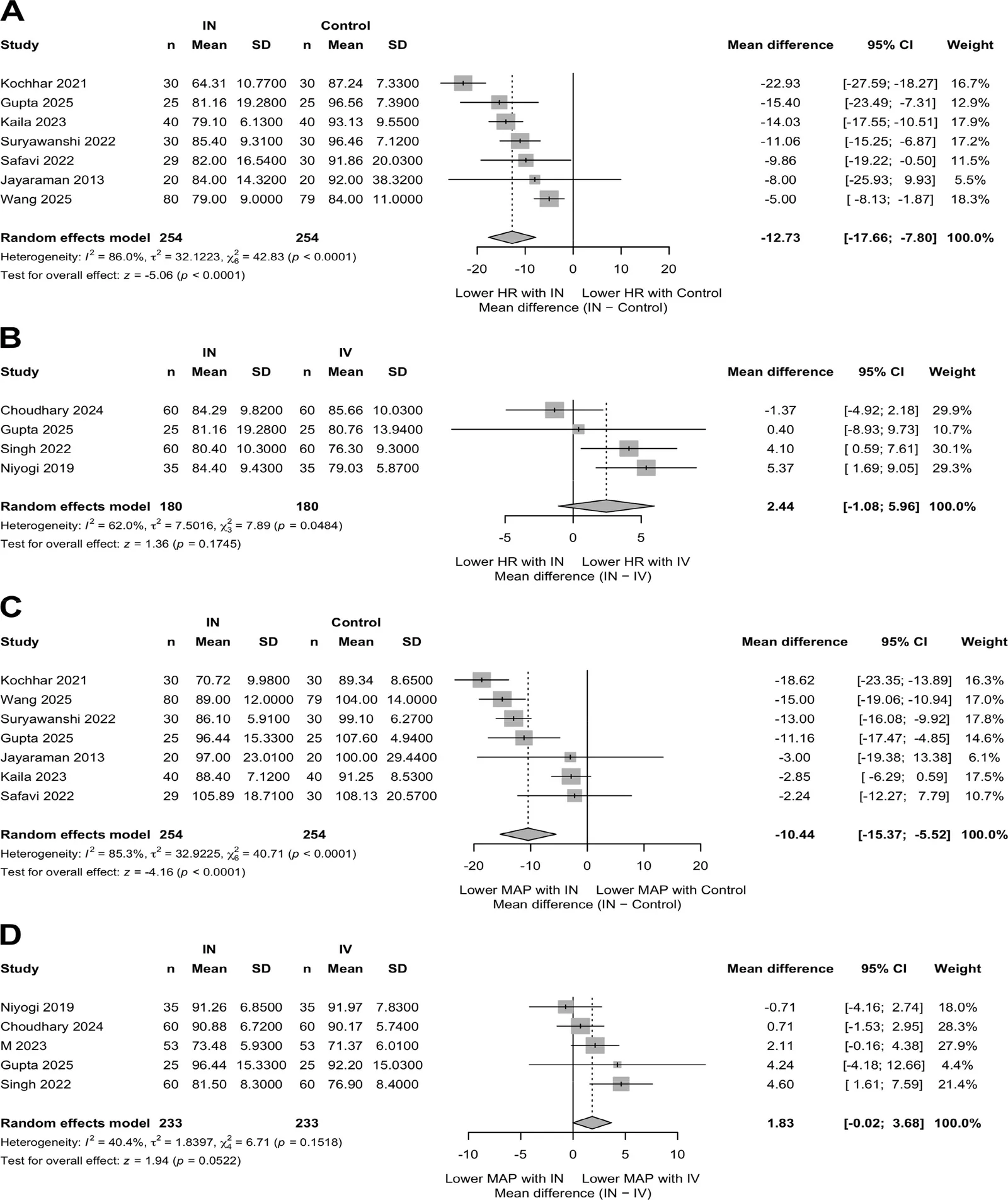
Forest plots for intraoperative hemodynamic outcomes comparing Intranasal dexmedetomidine (IN) to control or Intravenous dexmedetomidine (IV). (A) Heart rate at intubation: IN vs. control. (B) Heart rate at intubation: IN vs. IV. (C) Mean Arterial Pressure (MAP) at intubation: IN vs. control. (D) MAP at intubation: IN vs. IV. The effect measure calculated was mean difference. Negative values indicate lower HR or MAP with IN dexmedetomidine, whereas positive values indicate lower HR or MAP with the comparator.

Compared with control, IN dexmedetomidine administration was associated with deeper sedation in five studies (SMD = 0.76; 95% CI: 0.18 to 1.34, p = 0.010; *I*^2^ = 87.6%; Fig. 2A).^14^^,^^15^^,^^29^^,^^30^^,^^41^ Baseline HR and MAP did not differ statistically between groups. At intubation, seven studies demonstrated lower HR with IN dexmedetomidine versus control, although heterogeneity was substantial (MD = -12.73 bpm, 95% CI: -17.66 to -7.80, p < 0.001; *I*^2^ = 86%; Fig. 3A).^14^, ^15^, ^16^^,^^37^^,^^41^^,^^42^, ^43^, ^44^, ^45^ Similarly, MAP appeared lower with IN dexmedetomidine compared with control, with high heterogeneity (MD = -10.44 mmHg, 95% CI: -15.37 to -5.52, p < 0.001; *I*^2^ = 85%; Fig. 3C).^14^, ^15^, ^16^^,^^37^^,^^41^^,^^42^^,^^45^

At induction, HR and MAP were also lower with IN dexmedetomidine than control (HR MD = -13.37 bpm, p < 0.001; MAP MD = -10.65 mmHg, p < 0.001).^15^^,^^42^ Similar reductions were observed at 10 minutes into surgery (HR MD = -13.15 bpm, p < 0.001; MAP MD = -7.77 mmHg, p = 0.025).^16^^,^^37^^,^^42^^,^^45^

### Safety outcomes

The risk of bradycardia was similar between IN dexmedetomidine and control (RR = 1.15, 95% CI: 0.64 to 2.04, p = 0.645; *I*^2^ = 0%).^14^^,^^26^^,^^27^^,^^29^^,^^30^ Hypotension rates were comparable between IN dexmedetomidine and control (RR = 1.09, 95% CI: 0.61 to 1.96, p = 0.769; *I*^2^ = 7.9%).^14^^,^^26^^,^^27^^,^^29^, ^30^, ^31^

### Clinical effects of IN compared with IV dexmedetomidine

Compared with IV dexmedetomidine, IN administration was associated with lighter sedation in four studies (SMD = -0.54, 95% CI: -1.08 to -0.01, p = 0.047; *I*^2^ = 79%; Fig. 2B).^30^^,^^32^^,^^34^^,^^44^ Baseline HR and MAP did not differ statistically between groups. At intubation, no statistically significant differences in HR (MD = 2.44 bpm, 95% CI: -1.08 to 5.96, p = 0.174; *I*^2^ = 62%; Fig. 3B)^36^^,^^40^^,^^42^^,^^43^ or MAP (MD = 1.83 mmHg, 95% CI: -0.02 to 3.68, p = 0.052; *I*^2^ = 40%; Fig. 3D)^35^^,^^36^^,^^40^^,^^42^^,^^43^ were observed between IN and IV dexmedetomidine. No significant differences between IN and IV dexmedetomidine were observed at induction,^35^^,^^36^^,^^40^^,^^42^^,^^43^ nor at 10 minutes.^34^, ^35^, ^36^^,^^39^^,^^40^^,^^42^^,^^43^

### Safety outcomes

Bradycardia occurred significantly less frequently with IN compared with IV dexmedetomidine (RR = 0.31, 95% CI: 0.11 to 0.87, p = 0.026; *I*^2^ = 0%, Fig. S4).^30^^,^^32^^,^^36^^,^^44^ Limited data from two studies suggested a lower risk of hypotension with IN compared with IV dexmedetomidine administration (RR = 0.15, 95% CI: 0.03 to 0.79, p = 0.026).^32^^,^^36^

### Secondary outcomes

#### Propofol consumption

Across four studies comparing IN dexmedetomidine with control, three reported a significant reduction in propofol consumption in the IN group,^14^^,^^15^^,^^30^ while one study found no significant difference.^33^ When comparing IN with IV dexmedetomidine, one study demonstrated a significant decrease in the IV group,^30^ whereas another reported no significant change in propofol requirements.^39^

### 24-hour postoperative opioid consumption

One study comparing IN dexmedetomidine with control found no significant difference in postoperative opioid consumption.^30^ Conflicting findings were observed in studies that compared IN and IV dexmedetomidine. Namely, one study reported a significant increase (+12.9 μg sufentanil) in opioid consumption in the IN group,^30^ while another study demonstrated no significant difference between the two routes of administration.^34^

#### PACU time

Among studies comparing IN dexmedetomidine with control, two reported no significant difference in PACU length of stay,^14^^,^^37^ while one study demonstrated a significant reduction in PACU time (-6 minutes) in the IN group. ^31^ In comparisons between IN and IV dexmedetomidine, one study found no significant difference in PACU duration,^39^ whereas another reported a significant decrease in PACU time with IN administration.^44^

#### Postoperative nausea and vomiting

Among studies comparing IN dexmedetomidine with control, two studies reported no significant differences in PONV incidence between groups.^14^^,^^31^ In comparisons between IN and IV dexmedetomidine, two studies similarly demonstrated no significant differences in PONV outcomes.^36^^,^^44^ Additionally, one study comparing IN, IV, and control groups found no significant differences in PONV incidence in any of the treatment arms.^42^

### Sensitivity analyses

Leave-one-out sensitivity analyses showed that removing individual studies did not change the direction or significance for most outcomes. For outcomes that were borderline or non-significant, study removal produced minor shifts in effect size but did not alter the overall interpretation.

Heterogeneity across leave-one-out sensitivity analyses varied by outcome and comparison (Table S5). Exclusion of individual studies did not materially change the direction or statistical significance of pooled effects for most outcomes. However, the magnitude of between-study heterogeneity varied across outcomes and comparisons, as demonstrated in Table S5.

### Cochrane risk of bias assessment

Two studies were rated as having a low risk of bias,^27^^,^^33^ demonstrating robust methodological quality. The majority of studies were rated as having “some concerns”,^14^, ^15^, ^16^^,^^26^^,^^29^, ^30^, ^31^, ^32^^,^^34^, ^35^, ^36^^,^^39^, ^40^, ^41^, ^42^, ^43^, ^44^, ^45^ except one with a “high” overall risk of bias (Figs. S6‒S7).^37^ Most concerns were related to selection of reported results due to measurement of outcomes at multiple time points. Other less common concerns were related to randomization and deviations from intended interventions.

### GRADE certainty of evidence

The GRADE certainty of evidence across outcomes varied from very low to high. High-certainty evidence was observed for bradycardia and hypotension, while moderate-certainty evidence supported a subset of hemodynamic measures (Table S8). In contrast, sedation and several peri-intubation and post-induction hemodynamic outcomes were generally supported by low or very low certainty evidence. Downgrading most frequently resulted from inconsistency due to substantial between-study heterogeneity, with occasional downgrading for risk of bias.

## Discussion

This systematic review and meta-analysis of 25 RCTs evaluated preoperative IN dexmedetomidine in adult patients having inpatient surgery under GA across two separate pairwise comparisons: IN dexmedetomidine vs. control and IN vs. IV dexmedetomidine. Preoperative IN dexmedetomidine was associated with sedative and hemodynamic effects that may be clinically relevant, although certainty varied across outcomes. Compared with control, IN dexmedetomidine was associated with significantly deeper preoperative sedation and consistent attenuation of hemodynamic responses at induction, intubation, and in the early intraoperative period. Compared with IV dexmedetomidine, IN administration resulted in lighter sedation and broadly similar hemodynamic control, with lower observed rates of bradycardia and hypotension. These findings suggest that IN dexmedetomidine may provide effective preoperative sedation and hemodynamic modulation, although the safety comparison with IV administration is based on a limited number of trials and events and should be interpreted with caution.

Although the evidence in adults should be interpreted cautiously given heterogeneity and low certainty for sedation, the observed direction of effect is consistent with pediatric literature, where IN dexmedetomidine has been associated with high sedation success and greater sedative efficacy than midazolam.^12^^,^^13^^,^^48^ The sedative effects of dexmedetomidine are mediated primarily through selective activation of central α₂-adrenergic receptors, particularly within the locus coeruleus, leading to inhibition of norepinephrine release and reduced sympathetic outflow. This mechanism produces a state of cooperative sedation that resembles natural sleep, while preserving respiratory drive, a physiological profile that likely underlies its sedative effects across populations.^49^

Differences in route of administration and dosing kinetics provide a plausible explanation for the observed difference in sedative depth between IV and IN dexmedetomidine. Nevertheless, this difference may be clinically relevant, as deeper sedation has been associated with less favorable postoperative outcomes. One study reported longer time to awakening, delayed recovery, and prolonged PACU stays with IV administration compared with controls.^50^ These findings suggest that higher peak plasma concentrations with IV dexmedetomidine may lead to residual sedation. In contrast, IN administration results in slower absorption and lower peak concentrations, producing a more gradual and moderate sedative effect. This likely explains the lighter sedation observed in our study, while maintaining adequate sympatholytic and hemodynamic stability. Clinically, lighter preoperative sedation may be preferable when the primary goals are anxiolysis and attenuation of sympathetic responses rather than deep sedation, facilitating quicker recovery.

The hemodynamic findings are biologically plausible given the sympatholytic effects of dexmedetomidine and are consistent with prior orthopedic studies reporting reduced perioperative sympathetic responses with IN administration.^51^^,^^52^ Similar to sedative effects, this is likely explained by selective α₂-adrenergic receptor agonism in the locus coeruleus.^4^ In addition, presynaptic α₂-receptor activation inhibits peripheral norepinephrine release, further contributing to reductions in HR and MAP.^53^

There is no consensus definition for the magnitude of HR or MAP attenuation that constitutes a clinically significant blunting of the intubation response. In anesthetic practice, the reductions observed with IN dexmedetomidine relative to control, approximately 13 bpm in HR and 8 to 11 mmHg in MAP, may represent a modest attenuation of the sympathetic response rather than a clearly established patient-important benefit. Importantly, these reductions were not accompanied by increased bradycardia or hypotension, supporting their overall clinical acceptability. Their clinical relevance may be greatest in patients for whom sympathetic surges during intubation are undesirable.^54^

The apparent similarity in peri-induction hemodynamic control between IN and IV administration may reflect convergence in central sympatholytic effects despite differences in route and sedative depth. The absence of hemodynamic differences between routes may reflect a ceiling effect of α₂-mediated autonomic modulation during GA, whereby additional drug exposure does not translate into further measurable cardiovascular suppression.

Dexmedetomidine has some known adverse cardiovascular side effects, with hypotension and bradycardia being the most commonly reported.^52^ The apparent safety signal favoring IN administration is pharmacologically plausible given its slower systemic absorption and more gradual peak plasma concentrations. These safety estimates were derived from a small number of trials and few events, and should be regarded as preliminary rather than definitive evidence. Taken together with the lighter sedation observed with the IN route, these findings may suggest a possible efficacy-safety trade-off. A modest reduction in sedative depth may be an acceptable compromise when the priority is to avoid bradycardia and hypotension, particularly in patients in whom hemodynamic stability is critical. Confirmation in adequately powered safety trials is needed before this trade-off can be considered established.

### Study strengths and limitations

This study has several strengths. This was a comprehensive systematic review and meta-analysis restricted to RCTs, enhancing internal validity and minimizing confounding. Rigorous quantitative synthesis was complemented by sensitivity analyses, allowing exploration of heterogeneity and assessment of the robustness of pooled estimates. Certainty of evidence was systematically evaluated using the GRADE framework, providing transparent appraisal of confidence across outcomes. Finally, inclusion of a broad range of surgical populations enhances the generalizability of the findings to adult surgical patients, although this diversity may contribute to heterogeneity.

This study has several limitations. Although restricted to RCTs, some pooled outcomes were informed by a limited number of studies, reducing statistical power and precision. Substantial heterogeneity was observed for several outcomes, likely reflecting variability in IN dexmedetomidine dosing, timing of administration, surgical populations, and perioperative protocols, which contributed to low certainty of evidence for select endpoints. The predominance of single-country/single-region trials, variable quality of reporting, small sample sizes for several outcomes, differences in sedation scales, possible publication bias, and the limited ability to assess rare adverse events may limit the certainty and generalizability of these findings. Future research should focus on well-powered, multicenter RCTs to define optimal dosing, timing, and patient selection for IN dexmedetomidine, using standardized sedation and hemodynamic outcome measures to improve comparability across studies.

### Clinical implications

IN dexmedetomidine may represent an option for noninvasive premedication that provides sedation and peri-induction hemodynamic stability without a clear increase in adverse effects, particularly when IV access is limited or lighter sedation is desired. Its alignment with enhanced recovery after surgery (ERAS) principles supports consideration in adult preoperative care.

## Conclusion

Preoperative IN dexmedetomidine was associated with improved sedation and attenuation of peri-induction hemodynamic responses compared with controls. Compared with IV dexmedetomidine, IN administration showed broadly similar peri-induction hemodynamic effects and lower observed rates of bradycardia and hypotension, although these findings should be interpreted cautiously given heterogeneity and low or very low certainty for several outcomes. Overall, IN dexmedetomidine may represent a promising noninvasive premedication option in selected adult surgical patients, although confirmation in larger, standardized, adequately powered trials is still required.

## Data availability statement

The datasets generated and/or analyzed during the current study are available from the corresponding author upon reasonable request. The extracted aggregate-level dataset, analytic files, and statistical code supporting the findings of this systematic review and meta-analysis have been deposited in Mendeley Data and are available at: https://doi.org/10.17632/jsdtydr35v.1. The dataset is currently under embargo and will be made publicly available after the embargo period on September 4, 2026. No individual participant data were used. All data were extracted from published studies cited in the manuscript.

## Units

MAP was reported in mmHg and heart rate was reported in beats per minute (bpm).

## Declaration of AI and AI-assisted technologies

During the preparation of this manuscript, the authors used ChatGPT (OpenAI) to assist with language editing and refinement. The authors reviewed and edited all AI-assisted content and take full responsibility for the final manuscript.

## Author’s contribution

Vitaliy Voznyy: Conceptualization; Methodology; Data extraction; Drafted manuscript; Manuscript reviewing and editing. Mouad Elganga: Conceptualization; Methodology; Full-text screening; Statistical analyses; Drafted manuscript; Manuscript reviewing and editing. Leya Tawfik: Methodology; Screening; Data extraction; Drafted manuscript; Manuscript reviewing and editing. Tony Tan: Screening; Data extraction; Manuscript reviewing and editing. Sammie Yu: Data extraction; Manuscript reviewing and editing. Ellene Yan: Conceptualization; Methodology; Manuscript reviewing and editing. Aparna Saripella: Methodology; Manuscript reviewing and editing. Marina F. Englesakis: Designed and executed the database search strategies; Manuscript reviewing and editing. Frances Chung: Conceptualization; Methodology; Manuscript reviewing and editing.

## Funding

EY reports research support from the Canadian Institutes of Health Research (CIHR) Canada Graduate Scholarships Doctoral Award grant number: FBD-195197. AS reports research support from the University Health Network Foundation. FC reports research support from the Ontario Ministry of Health Innovation Grant, ResMed Foundation, University Health Network Foundation, and STOP-Bang Questionnaire proprietary to the University Health Network.

## Conflicts of interest

The authors declare no conflicts of interest.

## Appendix Supplementary materials

Image, application 1

## Footnotes

Supplementary material associated with this article can be found in the online version at doi:10.1016/j.bjane.2026.844820.
