# Supplementary material for: Efficacy and safety of preoperative intranasal dexmedetomidine in adults: a systematic review and meta-analysis of randomized controlled trials
Source: Braz J Anesthesiol. 2026 Sep 3;76(5):844820. doi: 10.1016/j.bjane.2026.844820 (PMC13594737; doi:10.1016/j.bjane.2026.844820)
Supplement: Supplementary file 1 [file mmc1.docx]

**BJAN-D-26-00166_Supplementary Material**

**Table of Contents**

[Table S1. MEDLINE Search Strategy	2](#_Toc221901693)

[Figure S2. IN vs Control Forest Plots	8](#_Toc221901694)

[Figure S3. IN vs IV Forest Plots	10](#_Toc221901695)

[Figure S4. Adverse Events Forest Plots	12](#_Toc221901696)

[Table S5. Leave-one-out Sensitivity Analysis Plots	14](#_Toc221901697)

[Figure S6. Risk of bias assessment of included studies	16](#_Toc221901698)

[Figure S7. Risk of bias assessment summary plot	17](#_Toc221901699)

[Figure S8. Summary of findings and certainty of evidence (GRADE)	18](#_Toc221901700)

[Table S9. PRISMA Checklist.	21](#_Toc221901701)

# **Table S1**. MEDLINE Search Strategy

**(1946 to November 26, 2025)**

| **#** | **Searches** | **Results** |
| --- | --- | --- |
| 1 | Dexmedetomidine/ | 6535 |
| 2 | dexmedetomidin??.mp,kw. | 8000 |
| 3 | dexmedetomidinum.mp,kw. | 0 |
| 4 | cepedex*.mp. | 0 |
| 5 | dexased*.mp. | 0 |
| 6 | dexmedesed*.mp. | 0 |
| 7 | dexmedetomidinehclc13h16n2hcl*.mp. | 0 |
| 8 | dexmedvet*.mp. | 0 |
| 9 | igalmi*.mp. | 3 |
| 10 | medetomidine*.mp. | 1773 |
| 11 | mpv 1440.mp,kw. | 2 |
| 12 | mpv-1440.mp,kw. | 2 |
| 13 | mpv1440.mp,kw. | 0 |
| 14 | Precedex???.mp,kw. | 40 |
| 15 | 67VB76HONO*.rn. [Dexmedetomidine] | 6527 |
| 16 | dextor??.mp,kw. | 0 |
| 17 | sc-78886.mp,kw. | 0 |
| 18 | 113775-47-6.mp,kw,rn. | 1 |
| 19 | dexdomitor??.mp,kw. | 7 |
| 20 | dexdor??.mp,kw. | 10 |
| 21 | primadex??.mp,kw. | 0 |
| 22 | sedadex*.mp. | 0 |
| 23 | sedexodine*.mp. | 0 |
| 24 | sileo.mp,kw. | 1 |
| 25 | or/1-24 [ Dexmedetomidine ] | 9418 |
| 26 | Administration, Intranasal/ | 17856 |
| 27 | intranasal drug administration/ | 17856 |
| 28 | (Nose/ or Nasal Cavity/ or Nasal Mucosa/) and Administration, Inhalation/ | 423 |
| 29 | (Nose/ or Nasal Cavity/ or Nasal Mucosa/) and Aerosols/ | 623 |
| 30 | (Nose/ or Nasal Cavity/ or Nasal Mucosa/) and Drug Delivery Systems/ | 550 |
| 31 | Nasal Sprays/ | 1104 |
| 32 | intranasal*.mp. | 37634 |
| 33 | intra-nasal*.mp. | 483 |
| 34 | (nasal* adj4 admin*).mp. | 3213 |
| 35 | (nasal* adj4 aerosol*).mp. | 333 |
| 36 | (nasal* and atomi*).mp. | 227 |
| 37 | (nasal* adj4 cavit*).mp. | 22832 |
| 38 | (nasal* adj4 deliver*).mp. | 1969 |
| 39 | (nasal* adj4 drop*).mp. | 607 |
| 40 | (nasal?? adj4 instill*).mp. | 387 |
| 41 | (nasal* adj4 irrigat*).mp. | 722 |
| 42 | (nasal* adj4 medicat*).mp. | 342 |
| 43 | (nasal* adj4 (mist or mists or misted or misting?)).mp. | 16 |
| 44 | (nasal* adj4 nebuli*).mp. | 133 |
| 45 | (nasal* adj4 spray*).mp. | 4213 |
| 46 | (nose? adj4 admin*).mp. | 190 |
| 47 | (nose? adj4 aerosol*).mp. | 135 |
| 48 | (nose? and atomi*).mp. | 101 |
| 49 | (nose? adj4 cavit*).mp. | 294 |
| 50 | (nose? adj4 deliver*).mp. | 719 |
| 51 | (nose? adj4 drop*).mp. | 296 |
| 52 | (nose? adj4 instill*).mp. | 34 |
| 53 | (nose? adj4 irrigat*).mp. | 23 |
| 54 | (nose? adj4 (mist or mists or misted or misting?)).mp. | 2 |
| 55 | (nose? adj4 nebuli*).mp. | 14 |
| 56 | (nose? adj3 spray*).mp. | 84 |
| 57 | or/26-56 [ Intranasal Administration ] | 64415 |
| 58 | 25 and 57 [ Dexmedetomidine + Intranasal Administration ] | 345 |
| 59 | exp Perioperative care/ | 165429 |
| 60 | exp Perioperative Nursing/ | 14177 |
| 61 | exp Perioperative Period/ | 110679 |
| 62 | exp Preoperative care/ | 75648 |
| 63 | perioperative period/ | 4420 |
| 64 | preoperative education/ | 0 |
| 65 | preoperative evaluation/ | 0 |
| 66 | preoperative treatment/ | 0 |
| 67 | preoperative complication/ | 0 |
| 68 | exp peroperative care/ | 0 |
| 69 | exp peroperative complication/ | 59851 |
| 70 | Surgical Clearance/ | 51 |
| 71 | (ahead adj3 intervention?).mp. | 79 |
| 72 | (ahead adj3 operat????).mp. | 29 |
| 73 | (ahead adj3 procedur*).mp. | 28 |
| 74 | (ahead adj3 surgery).mp. | 77 |
| 75 | (ahead adj3 surgical*).mp. | 22 |
| 76 | (an?esth* adj4 clinic?).mp,kw. | 880 |
| 77 | (an?esth* adj3 consult*).mp. | 814 |
| 78 | (an?esth* adj4 counsel*).mp,kw. | 70 |
| 79 | (an?esth* adj3 induct*).mp. | 17825 |
| 80 | (before adj3 induct*).mp. | 9725 |
| 81 | (before adj2 operat????).mp. | 18539 |
| 82 | (before adj2 surgery).mp. | 55957 |
| 83 | (before adj3 procedur*).mp. | 12320 |
| 84 | (clear* adj3 (surgery or surgeries or surgical*)).mp. | 3788 |
| 85 | (fit* adj3 (surgery or surgeries or surgical*)).mp. | 1368 |
| 86 | (in advance adj3 intervention?).mp. | 66 |
| 87 | (in advance adj3 operat*).mp. | 55 |
| 88 | (in advance adj3 procedur*).mp. | 65 |
| 89 | (in advance adj3 surger*).mp. | 94 |
| 90 | (in advance adj3 surgical*).mp. | 38 |
| 91 | ((patient?? or inpatient??) adj4 (fit or fitness)).mp. | 7330 |
| 92 | ((patient?? or inpatient??) adj4 (suitable* or suitabilit*)).mp. | 14963 |
| 93 | ((patient?? or inpatient??) adj8 (workup* or work-up*)).mp. | 12273 |
| 94 | (preced* adj3 intervention?).mp. | 456 |
| 95 | (preced* adj3 operat*).mp. | 416 |
| 96 | (preced* adj3 procedur*).mp. | 568 |
| 97 | (preced* adj3 surger*).mp. | 989 |
| 98 | (preced* adj3 surgical*).mp. | 325 |
| 99 | (preceeding adj3 intervention?).mp. | 2 |
| 100 | (preceeding adj3 operat*).mp. | 6 |
| 101 | (preceeding adj3 procedur*).mp. | 6 |
| 102 | (preceeding adj3 surg*).mp. | 11 |
| 103 | (preceeding adj3 surger*).mp. | 7 |
| 104 | (prior adj2 operat????).mp. | 2633 |
| 105 | (prior adj2 surgery).mp. | 19651 |
| 106 | (prior adj3 procedur*).mp. | 5066 |
| 107 | "prior to".mp. | 672705 |
| 108 | perian?esthe*.mp. | 682 |
| 109 | peri-an?esthe*.mp. | 104 |
| 110 | perioperat*.mp. | 137440 |
| 111 | peri-operat*.mp. | 8889 |
| 112 | periprocedur*.mp. | 7502 |
| 113 | peri-procedur*.mp. | 1695 |
| 114 | peri-surg*.mp. | 83 |
| 115 | perisurg*.mp. | 141 |
| 116 | peroperat*.mp. | 4950 |
| 117 | preanaesth*.mp. | 323 |
| 118 | pre-anaesth*.mp. | 397 |
| 119 | preanesth*.mp. | 9040 |
| 120 | pre-anesth*.mp. | 572 |
| 121 | pregeneral.mp. | 2 |
| 122 | pre-general.mp. | 5 |
| 123 | preintervention*.mp. | 5594 |
| 124 | pre-intervention*.mp. | 7188 |
| 125 | premedicat*.mp. | 20661 |
| 126 | pre-medicat*.mp. | 560 |
| 127 | preoperat*.mp. | 388623 |
| 128 | pre-operat*.mp. | 38404 |
| 129 | preoperat*.mp. | 388623 |
| 130 | pre-operat*.mp. | 38404 |
| 131 | preprocedur*.mp. | 5137 |
| 132 | pre-procedur*.mp. | 3521 |
| 133 | presurgery.mp. | 1688 |
| 134 | pre-surgery.mp. | 2055 |
| 135 | presurgical*.mp. | 10263 |
| 136 | pre-surgical*.mp. | 3677 |
| 137 | pretransplant*.mp. | 10302 |
| 138 | pre-transplant*.mp. | 5369 |
| 139 | "before undergo*".mp. | 2793 |
| 140 | "before having".mp. | 475 |
| 141 | "present* for surgery".mp. | 1613 |
| 142 | or/59-141 [ Preoperative & related terms ] | 1495865 |
| 143 | 58 and 142 [ Dexmedetomidine + Intranasal Administration + Preoperative ] | 153 |
| 144 | limit 143 to english language | 148 |
| 145 | remove duplicates from 144 | 147 |
| 146 | 145 not ((clinical conference or clinical trial, veterinary or clinical trial protocol or consensus development conference or consensus development conference, NIH or news or newspaper article or study guide or preprint).pt. or (arxiv or medrxiv or biorxiv or research square).so. or chapter.pr. or NB*.bk.) | 142 |
| 147 | 146 not (exp animals/ not (exp animals/ and (exp humans/ or exp patients/))) | 136 |
| 148 | limit 146 to humans | 136 |
| 149 | 147 or 148 [ limit to humans ] | 136 |
| 150 | limit 149 to ("all infant (birth to 23 months)" or "all child (0 to 18 years)" or "newborn infant (birth to 1 month)" or "infant (1 to 23 months)" or "preschool child (2 to 5 years)" or "child (6 to 12 years)" or "adolescent (13 to 18 years)") | 110 |
| 151 | 149 not 150 | 26 |
| 152 | limit 149 to ("all adult (19 plus years)" or "young adult (19 to 24 years)" or "adult (19 to 44 years)" or "young adult and adult (19-24 and 19-44)" or "middle age (45 to 64 years)" or "middle aged (45 plus years)" or "all aged (65 and over)" or "aged (80 and over)") | 29 |
| 153 | 151 or 152 [ limiting to adults ] | 33 |
| 154 | 153 [ Dexmedetomidine + Intranasal Administration + Preoperative; limits applied ] | 33 |

# **Figure S2.** IN vs Control Forest Plots

Forest plots for hemodynamics (heart rate [HR] and mean arterial pressure [MAP]) comparing intranasal (IN) dexmedetomidine with controls. (A) HR at baseline; (B) HR at induction; (C) HR at 10 minutes into surgery; (D) MAP at baseline; (E) MAP at induction; (F) MAP at 10 minutes into surgery. The effect measure calculated was mean difference.

# Figure S3. IN vs IV Forest Plots

Forest plots for hemodynamics (heart rate [HR] and mean arterial pressure [MAP]) comparing intranasal (IN) dexmedetomidine with intravenous (IV) dexmedetomidine. (A) HR at baseline; (B) HR at induction; (C) HR at 10 minutes into surgery; (D) MAP at baseline; (E) MAP at induction; (F) MAP at 10 minutes into surgery. The effect measure calculated was mean difference.

# **Figure S4.** Adverse Events Forest Plots

Forest plots for intraoperative hemodynamic outcomes comparing intranasal dexmedetomidine (IN) to control or intravenous dexmedetomidine (IV). (A) Hypotension risk of IN vs. control; (B) Hypotension risk IN vs. IV; (C) Bradycardia risk IN vs. control; (D) Bradycardia risk IN vs. IV. The effect measure calculated was risk ratio.

# **Table S5**. Leave-one-out Sensitivity Analysis Plots

| **Outcome** | Effect Measure | n | Base I^2^ | I^2^ Minimum | I^2^ Maximum | Maximum I^2^ Change | Study corresponding to Max I^2^ Change |
| --- | --- | --- | --- | --- | --- | --- | --- |
| Sedation score post-medication (SMD; harmonized): IN vs Control | SMD | 5 | 85.7 | 67.8 | 89.6 | 17.8 | Lu 2016 |
| Sedation score post-medication (SMD; harmonized): IN vs IV | SMD | 4 | 80.3 | 0 | 87.5 | 80.3 | Mohammed 2024 |
| Heartrate at Baseline (MD): IN vs Control | MD | 8 | 45.9 | 0 | 57.7 | 45.9 | Kaila 2023 |
| Heartrate at Baseline (MD): IN vs IV | MD | 6 | 66.6 | 42.8 | 73 | 23.8 | M 2023 |
| Heartrate at Induction (MD): IN vs IV | MD | 5 | 14.5 | 0 | 30.7 | 16.2 | Choudhary 2024 |
| Heartrate at Intubation (MD): IN vs Control | MD | 7 | 83.4 | 65.8 | 86.7 | 17.6 | Kochhar 2021 |
| Heartrate at Intubation (MD): IN vs IV | MD | 4 | 62.1 | 0 | 74 | 62.1 | Choudhary 2024 |
| Heartrate at 10_mins (MD): IN vs Control | MD | 4 | 75.6 | 0 | 85 | 75.6 | Suryawanshi 2022 |
| Heartrate at 10_mins (MD): IN vs IV | MD | 6 | 85.2 | 77.6 | 88.9 | 7.6 | Kohaf 2024 |
| MAP at Baseline (MD): IN vs Control | MD | 7 | 48.1 | 9 | 55.9 | 39.1 | Suryawanshi 2022 |
| MAP at Baseline (MD): IN vs IV | MD | 7 | 51.5 | 31.8 | 58.5 | 19.7 | Singh 2022 |
| MAP at Induction (MD): IN vs IV | MD | 4 | 0 | 0 | 0 | 0 | Singh 2022 |
| MAP at Intubation (MD): IN vs Control | MD | 7 | 84.6 | 63.9 | 88.1 | 20.7 | Kaila 2023 |
| MAP at Intubation (MD): IN vs IV | MD | 5 | 43.9 | 0 | 57.3 | 43.9 | Singh 2022 |
| MAP at 10_mins (MD): IN vs Control | MD | 4 | 87.9 | 0 | 93.2 | 87.9 | Gupta 2025 |
| MAP at 10_mins (MD): IN vs IV | MD | 7 | 73.5 | 65.5 | 78.3 | 8 | Singh 2022 |
| Bradycardia (RR): IN vs Control | RR | 5 | 0 | 0 | 0 | 0 | Wang 2025 |
| Hypotension (RR): IN vs Control | RR | 5 | 0 | 0 | 0 | 0 | Qiao 2016 |
| Bradycardia (RR): IN vs IV | RR | 4 | 0 | 0 | 0 | 0 | Wu 2016 |

^Leave-one-out sensitivity analyses were used to assess the influence of individual studies on between-study heterogeneity and the pooled estimate. “Base I²” is the heterogeneity from the primary random-effects model including all studies; “I² Minimum” and “I² Maximum” are the lowest and highest I² observed after omitting each study one at a time; “Maximum I² Change” is the largest absolute deviation in I² from the base model, and the corresponding omitted study is listed in the final column. Values are shown only for outcomes with ≥ 3 studies.^

# **Figure S6**. Risk of bias assessment of included studies

# **Figure S7**. Risk of bias assessment summary plot

As percentage (intention-to-treat)

|  | Low risk | Some concerns | High risk |
| --- | --- | --- | --- |
| Randomization process | 76.2 | 19 | 4.8 |
| Deviations from intended interventions | 85.7 | 14.3 | 0 |
| Mising outcome data | 100 | 0 | 0 |
| Measurement of the outcome | 100 | 0 | 0 |
| Selection of the reported result | 19 | 81 | 0 |
| Overall Bias | 9.5 | 85.7 | 4.8 |

# **Figure S8**. Summary of findings and certainty of evidence (GRADE)

| **Outcome** | **Effect on IN Group**  **(95% CI)** | **No. of Participants (Studies)** | **Risk of Bias** | **Imprecision** | **Inconsistency** | **Indirectness** | **Publication Bias** | **GRADE Certainty of Evidence** |
| --- | --- | --- | --- | --- | --- | --- | --- | --- |
| Sedation Score (IN vs Control) | SMD **0.76 Higher**  (0.18-1.34) | 393 (5 RCTs) | Not Serious | Not Serious | Very serious^a^ | Not Serious | Not Serious | ⨁⨁◯◯  Low^a^ |
| Sedation Score (IN vs IV) | SMD **0.54 Lower**  (-1.08-0.01) | 303 (4 RCTs) | Not Serious | Not Serious | Very serious^a^ | Not Serious | Not Serious | ⨁⨁◯◯  Low^a^ |
| Baseline HR (IN vs Control) | MD **0.38 Lower**  (-2.57-1.80) | 568 (8 RCTs) | Serious^b^ | Not Serious | Serious^c^ | Not Serious | Not Serious | ⨁⨁◯◯  Low^b, c^ |
| Baseline HR (IN vs IV) | MD **0.14 Lower**  (-3.41-3.14) | 495 (6 RCTs) | Serious^b^ | Not Serious | Serious^c^ | Not Serious | Not Serious | ⨁⨁◯◯  Low^b, c^ |
| HR at Induction (IN vs Control) | MD **13.37 Lower**  (-18.36-  -8.39) | 110 (2 RCTs) | Not Serious | Not Serious | Not Serious | Not Serious | Not Serious | ⨁⨁⨁⨁  High |
| HR at Induction (IN vs IV) | MD **1.41 Higher**  (-0.36-3.17) | 466 (5 RCTs) | Not Serious | Not Serious | Not Serious | Not Serious | Not Serious | ⨁⨁⨁⨁  High |
| HR at Intubation (IN vs Control) | MD **12.73**  **Lower**  (-17.66-  7.80) | 508 (7 RCTs) | Serious^b^ | Not Serious | Very Serious^a^ | Not Serious | Not Serious | ⨁◯◯◯  Very Low^a, b^ |
| HR at Intubation (IN vs IV) | MD **2.44 Higher**  (-1.08-5.96) | 360 (4 RCTs) | Not Serious | Not Serious | Serious^c^ | Not Serious | Not Serious | ⨁⨁⨁◯  Moderate^c^ |
| HR at 10 Minutes (IN vs Control) | MD **13.15 Lower**  (-18.02-  -8.27) | 149 (4 RCTs) | Serious^b^ | Not Serious | Very Serious^a^ | Not Serious | Not Serious | ⨁⨁◯◯  Low^a^ |
| HR at 10 Minutes (IN vs IV) | MD **4.09 Higher**  (-0.18-8.37) | 449 (6 RCTs) | Not Serious | Not Serious | Very Serious^a^ | Not Serious | Not Serious | ⨁⨁◯◯  Low^a^ |
| Baseline MAP (IN vs Control) | MD **1.05 Higher**  (-0.97-3.08) | 508 (7 RCTs) | Not Serious | Not Serious | Serious^c^ | Not Serious | Not Serious | ⨁⨁⨁◯  Moderate^c^ |
| Baseline MAP (IN vs IV) | MD **0.10 Higher**  (-2.17-2.37) | 555 (7 RCTs) | Not Serious | Not Serious | Serious^c^ | Not Serious | Not Serious | ⨁⨁⨁◯  Moderate^c^ |
| MAP at Induction (IN vs Control) | MD **10.65 Lower**  (-16.56-  -4.75) | 110 (2 RCTs) | Not Serious | Not Serious | Serious^c^ | Not Serious | Not Serious | ⨁⨁⨁◯  Moderate^c^ |
| MAP at Induction (IN vs IV) | MD **0.81 Lower**  (-2.44-0.82) | 360 (4 RCTs) | Not Serious | Not Serious | Not Serious | Not Serious | Not Serious | ⨁⨁⨁⨁  High |
| MAP at Intubation (IN vs Control) | MD **10.44**  **Lower**  (-15.37-  -5.52) | 508 (7 RCTs) | Serious^b^ | Not Serious | Very Serious^a^ | Not Serious | Not Serious | ⨁◯◯◯  Very Low^a, b^ |
| MAP at Intubation (IN vs IV) | MD **1.83 Higher**  (-0.02-3.68) | 466 (5 RCTs) | Not Serious | Not Serious | Serious^c^ | Not Serious | Not Serious | ⨁⨁⨁◯  Moderate^c^ |
| MAP at 10 Minutes (IN vs Control) | MD **7.77**  **Lower**  (-14.56-  -0.98) | 249 (4 RCTs) | Serious^b^ | Not Serious | Very Serious^a^ | Not Serious | Not Serious | ⨁◯◯◯  Very Low^a, b^ |
| MAP at 10 Minutes (IN vs IV) | MD **0.69**  **Higher**  (-1.80-3.18) | 555 (7 RCTs) | Not Serious | Not Serious | Serious^c^ | Not Serious | Not Serious | ⨁⨁⨁◯  Moderate^c^ |
| Bradycardia Incidence (IN vs Control) | RR **1.15**  (0.64-2.04) | 433 (5 RCTs) | Not Serious | Not Serious | Not Serious | Not Serious | Not Serious | ⨁⨁⨁⨁  High |
| Bradycardia Incidence (IN vs IV) | RR **0.31**  (0.11-0.87) | 363 (4 RCTs) | Not Serious | Not Serious | Not Serious | Not Serious | Not Serious | ⨁⨁⨁⨁  High |
| Hypotension Incidence (IN vs Control) | RR **1.09**  (0.61-1.96) | 493 (6 RCTs) | Not Serious | Not Serious | Not Serious | Not Serious | Not Serious | ⨁⨁⨁⨁  High |
| Hypotension Incidence (IN vs IV) | RR **0.15**  (0.03-0.79) | 293 (3 RCTs) | Not Serious | Not Serious | Not Serious | Not Serious | Not Serious | ⨁⨁⨁⨁  High |

**Explanations**

a. > 75% heterogeneity.
b. High risk of bias in at least one of the studies involved in the pooled analysis.
c. > 40% heterogeneity.

# **Table S9**. PRISMA Checklist.

| **Section and Topic** | **Item #** | **Checklist Item** | **Location Where Item is Reported** |
| --- | --- | --- | --- |
| **TITLE** | | |  |
| Title | 1 | Identify the report as a systematic review. | Pg 1 |
| **ABSTRACT** | | |  |
| Abstract | 2 | See the PRISMA 2020 for Abstracts checklist. | Pg 2-3 |
| **INTRODUCTION** | | |  |
| Rationale | 3 | Describe the rationale for the review in the context of existing  knowledge. | Pg 2 |
| Objectives | 4 | Provide an explicit statement of the objective(s) or question(s) the review addresses. | Pg 2 |
| **METHODS** | | |  |
| Eligibility  criteria | 5 | Specify the inclusion and exclusion criteria for the review and how studies were grouped for the syntheses. | Pg 6 |
| Information sources | 6 | Specify all databases, registers, websites, organisations, reference lists and other sources searched or consulted to identify studies. Specify the date when each source was last searched or consulted. | Pg 5 |
| Search strategy | 7 | Present the full search strategies for all databases, registers and websites, including any filters and limits used. | Pg 2, 5-6, Table S3 |
| Selection process | 8 | Specify the methods used to decide whether a study met the inclusion criteria of the review, including how many reviewers screened each record and each report retrieved, whether they worked independently, and if applicable, details of automation tools used in the process. | Pg 6 |
| Data collection process | 9 | Specify the methods used to collect data from reports, including how many reviewers collected data from each report, whether they worked independently, any processes for obtaining or confirming data from study investigators, and if applicable, details of automation tools used in the process. | Pg 7 |
| Data items | 10a | List and define all outcomes for which data were sought. Specify whether all results that were compatible with each outcome domain in each study were sought (e.g. for all measures, time points, analyses), and if not, the methods used to decide which results to collect. | Pg 7 |
|  | 10b | List and define all other variables for which data were sought (e.g. participant and intervention characteristics, funding sources). Describe any assumptions made about any missing or unclear information. | Pg 7 |
| Study risk of bias assessment | 11 | Specify the methods used to assess risk of bias in the included studies, including details of the tool(s) used, how many reviewers assessed each study and whether they worked independently, and if applicable, details of automation tools used in the process. | Pg 7 |
| Effect measures | 12 | Specify for each outcome the effect measure(s) (e.g. risk ratio, mean difference) used in the synthesis or presentation of results. | Pg 7-8 |
| Synthesis methods | 13a | Describe the processes used to decide which studies were eligible for each synthesis (e.g. tabulating the study intervention characteristics and comparing against the planned groups for each synthesis (item #5)). | Pg 7-8 |
|  | 13b | Describe any methods required to prepare the data for presentation or synthesis, such as handling of missing summary statistics, or data conversions. | Pg 7-8 |
|  | 13c | Describe any methods used to tabulate or visually display results of individual studies and syntheses. | Pg 7-8 |
|  | 13d | Describe any methods used to synthesize results and provide a rationale for the choice(s). If meta-analysis was performed, describe the model(s), method(s) to identify the presence and extent of statistical heterogeneity, and software package(s) used. | Pg 7-8 |
|  | 13e | Describe any methods used to explore possible causes of heterogeneity among study results (e.g. subgroup analysis, meta-regression). | Pg 7-8 |
|  | 13f | Describe any sensitivity analyses conducted to assess robustness of the synthesized results. | Pg 8 |
| Reporting bias assessment | 14 | Describe any methods used to assess risk of bias due to missing results in a synthesis (arising from reporting biases). | Pg 7 |
| Certainty assessment | 15 | Describe any methods used to assess certainty (or confidence) in the body of evidence for an outcome. | Pg 8 |
| **RESULTS** | | |  |
| Study selection | 16a | Describe the results of the search and selection process, from the number of records identified in the search to the number of studies included in the review, ideally using a flow diagram. | Pg 8-9 |
|  | 16b | Cite studies that might appear to meet the inclusion criteria, but which were excluded, and explain why they were excluded. | Pg 8-9 |
| Study characteristics | 17 | Cite each included study and present its characteristics. | Pg 8, Table 1 |
| Risk of bias in studies | 18 | Present assessments of risk of bias for each included study. | Pg 14 |
| Results of individual studies | 19 | For all outcomes, present, for each study: (a) summary statistics for each group (where appropriate) and (b) an effect estimate and its precision (e.g. confidence/credible interval), ideally using structured tables or plots. | Pg 10-13, Figure 2, Figure 3 |
| Results of Syntheses | 20a | For each synthesis, briefly summarise the characteristics and risk of bias among contributing studies. | Pg 10-13 |
|  | 20b | Present results of all statistical syntheses conducted. If meta-analysis was done, present for each the summary estimate and its precision (e.g. confidence/credible interval) and measures of statistical heterogeneity. If comparing groups, describe the direction of the effect. | Pg 10-13 |
|  | 20c | Present results of all investigations of possible causes of heterogeneity among study results. | Pg 13-14 |
|  | 20d | Present results of all sensitivity analyses conducted to assess the robustness of the synthesized results. | Pg 13-14 |
| Reporting biases | 21 | Present assessments of risk of bias due to missing results (arising from reporting biases) for each synthesis assessed. | Pg 12 |
| Certainty of evidence | 22 | Present assessments of certainty (or confidence) in the body of evidence for each outcome assessed. | Pg 13 |
| **DISCUSSION** | | |  |
| Discussion | 23a | Provide a general interpretation of the results in the context of other evidence. | Pg 15-16 |
|  | 23b | Discuss any limitations of the evidence included in the review. | Pg 19 |
|  | 23c | Discuss any limitations of the review processes used. | Pg 19 |
|  | 23d | Discuss implications of the results for practice, policy, and future research. | Pg 19-20 |
| **OTHER INFORMATION** | | |  |
| Registration and protocol | 24a | Provide registration information for the review, including register name and registration number, or state that the review was not registered. | Pg 5 |
|  | 24b | Indicate where the review protocol can be accessed, or state that a protocol was not prepared. | Pg 5 |
|  | 24c | Describe and explain any amendments to information provided at registration or in the protocol. | N/A |
| Sources | 25 | Describe sources of financial or non-financial support for the review, and the role of the funders or sponsors in the review. | Pg 1 |
| Competing interests | 26 | Declare any competing interests of review authors. | Pg 1 |
| Availability of data, code and other materials | 27 | Report which of the following are publicly available and where they can be found: template data collection forms; data extracted from included studies; data used for all analyses; analytic code; any other materials used in the review. | N/A |
